# A role of TARPs in the expression and plasticity of calcium-permeable AMPARs: Evidence from cerebellar neurons and glia

**DOI:** 10.1016/j.neuropharm.2013.03.037

**Published:** 2013-11

**Authors:** Cécile Bats, Mark Farrant, Stuart G. Cull-Candy

**Affiliations:** Department of Neuroscience, Physiology and Pharmacology, University College London, Gower Street, London WC1E 6BT, UK

**Keywords:** Cerebellum, Synaptic transmission, Plasticity, Glutamate receptors, AMPA receptors, Calcium-permeable AMPA receptors, TARPs

## Abstract

The inclusion of GluA2 subunits has a profound impact on the channel properties of AMPA receptors (AMPARs), in particular rendering them impermeable to calcium. While GluA2-containing AMPARs are the most abundant in the central nervous system, GluA2-lacking calcium-permeable AMPARs are also expressed in wide variety of neurons and glia. Accumulating evidence suggests that the dynamic control of the GluA2 content of AMPARs plays a critical role in development, synaptic plasticity, and diverse neurological conditions ranging from ischemia-induced brain damage to drug addiction. It is thus important to understand the molecular mechanisms involved in regulating the balance of AMPAR subtypes, particularly the role of their co-assembled auxiliary subunits. The discovery of transmembrane AMPAR regulatory proteins (TARPs), initially within the cerebellum, has transformed the field of AMPAR research. It is now clear that these auxiliary subunits play a key role in multiple aspects of AMPAR trafficking and function in the brain. Yet, their precise role in AMPAR subtype-specific regulation has only recently received particular attention. Here we review recent findings on the differential regulation of calcium-permeable (CP-) and -impermeable (CI-) AMPARs in cerebellar neurons and glial cells, and discuss the critical involvement of TARPs in this process.

This article is part of the Special Issue entitled ‘Glutamate Receptor-Dependent Synaptic Plasticity’.

## Highlights

• Calcium-permeable AMPARs are present in various cerebellar neurons and glial cells.
• The contribution of calcium-permeable AMPARs to transmission is dynamically regulated.
• TARPs influence the relative expression of AMPAR subtypes.
• Evidence suggests that TARPs play a role in calcium-permeable AMPAR plasticity.

## 1 Introduction

Many properties of AMPARs are dictated by the edited GluA2 subunit (Geiger et al., 1995; Swanson et al., 1997). AMPARs without GluA2 are permeable to calcium and display an inwardly rectifying IV-relationship, as they are blocked by endogenous intracellular polyamines at positive potentials, (Bowie and Mayer, 1995; Kamboj et al., 1995; Koh et al., 1995). When compared with their GluA2-containing counterparts, CP-AMPARs display a greater channel conductance (Feldmeyer et al., 1999; Swanson et al., 1997) and faster kinetics (Geiger et al., 1995). Expression, assembly and trafficking of CP-AMPARs are essential to basal transmission at many central synapses, and play pivotal roles in several important forms of synaptic plasticity. At the same time, over activation of these receptors can be injurious, and is thought to be a major contributor to cell death following stroke and hypoxic–ischemic white matter damage in infants. In addition, the upregulation or dysfunction of CP-AMPARs appears to be a significant contributor in several neurological disease states including glioblastoma cell proliferation, chronic pain and drug addiction (Cull-Candy et al., 2006; Kwak and Weiss, 2006; Liu and Zukin, 2007). For these reasons there has been growing interest in the regulation and plasticity of CP-AMPARs.

**Fig. 1:**
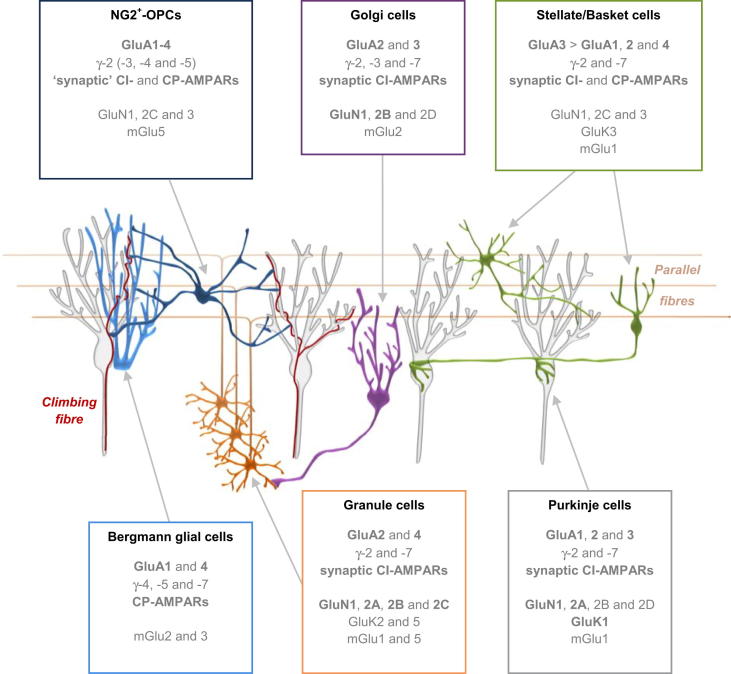
Main cerebellar neurons and glial cells: location, connectivity, glutamate receptor and TARP content. Subunits indicated in bold form a majority of the AMPARs, NMDARs, and KARs involved in fast synaptic transmission. Other subunits listed are also expressed, but evidence of their contribution to synaptic currents is unclear. Note that extrasynaptic receptors can be activated by glutamate spillover, and that mGluRs are usually located perisynaptically.

It has become clear that the diversity of native AMPAR properties is determined not only by AMPAR subunit composition and posttranslational modifications (such as phosphorylation; Lu and Roche, 2012), but also by the presence of auxiliary AMPAR subunits. Following the recognition that stargazin (γ-2) is a key regulator of AMPAR behaviour (Chen et al., 2000; Hashimoto et al., 1999), a number of related transmembrane AMPAR regulatory proteins have been identified (TARPs γ-3, -4, -5, -7, and -8) (Kato et al., 2007; Soto et al., 2009; Tomita et al., 2003). These various TARPs differ in their influence on AMPAR properties and display distinct, although partially overlapping, patterns of expression in the cerebellum (see Fig. 1) and elsewhere in the CNS (Fukaya et al., 2005; Tomita et al., 2003). Native AMPARs are thought to contain from one to four TARPs in addition to their core pore-forming subunits (Hastie et al., 2013; Kim et al., 2010; Shi et al., 2009); however, it is generally thought that only one type of TARP is present within a given AMPAR complex (Kato et al., 2007; Tomita et al., 2003). TARP association modifies several important aspects of AMPAR function. It increases their single-channel conductance (Soto et al., 2007, 2009; Tomita et al., 2005a), slows their deactivation and desensitization (Bedoukian et al., 2006; Cho et al., 2007; Korber et al., 2007; Milstein et al., 2007; Priel et al., 2005; Tomita et al., 2005a; Turetsky et al., 2005), attenuates voltage-dependent block by endogenous intracellular polyamines and modifies their pharmacological properties (Korber et al., 2007; Soto et al., 2007, 2009; Turetsky et al., 2005).

TARPs also play a critical role in AMPAR trafficking, promoting AMPAR maturation (Vandenberghe et al., 2005), delivery to the cell surface and clustering at the synapse (Chen et al., 2000; Kato et al., 2007; Soto et al., 2009; Tomita et al., 2003; Vandenberghe et al., 2005). Recent evidence also suggests that TARPs are involved in the regulation of AMPAR number that occurs with long-term potentiation (LTP) or depression (LTD) of synaptic transmission in hippocampal pyramidal neurons (Tomita et al., 2005b) and in cerebellar Purkinje cells (Nomura et al., 2012). While the role of TARPs in the neuronal trafficking of GluA2-containing CI-AMPARs is relatively well characterized, their role in the regulation of CP-AMPAR expression is much less well understood.

The importance of TARPs for AMPAR expression and function was revealed initially in the cerebellum, where the lack of γ-2 in the mutant mice *waggler* and *stargazer* (*stg*/*stg*) was associated with a selective loss of AMPAR-mediated synaptic currents in cerebellar granule cells (Chen et al., 2000; Hashimoto et al., 1999; Tomita et al., 2003). Whereas granule cells contain only CI-AMPARs, a variety of other cerebellar neurons and glia express both CP- and CI-AMPARs (see Fig. 1). Recent studies conducted on the cerebellum of *stg*/*stg* mice indicate that the extent of the disruption to AMPAR-mediated currents caused by the absence of γ-2 varies from one cell type to another, and depends both on the other TARP isoforms normally expressed, as well as the subtypes of AMPARs present (Bats et al., 2012; Jackson and Nicoll, 2011; Menuz et al., 2008; Yamazaki et al., 2010). There is now growing evidence for a differential regulation of CI- and CP-AMPARs by TARPs (Bats et al., 2012; Soto et al., 2007, 2009; Yamazaki et al., 2010; Zonouzi et al., 2011). Below we present recent findings and discuss the specific roles of γ-2 and other TARPs in the regulation of CP-AMPAR expression and plasticity.

## 2 CP-AMPARs in the cerebellum

### 2.1 CP-AMPARs in molecular layer interneurons: stellate and basket cells

The cerebellar cortex plays an essential role in the learning and execution of coordinated movements. Stellate and basket cells – inhibitory molecular layer interneurons – influence the output of the cerebellar cortex by modulating the spatiotemporal activity of Purkinje cells (Dizon and Khodakhah, 2011; Häusser and Clark, 1997; Wulff et al., 2009). While stellate cells are found primarily in the outer region of the molecular layer (where they form synapses with Purkinje cell dendrites), basket cells are found in the inner molecular layer and make characteristic perisomatic synaptic contacts with Purkinje cells (Fig. 1). The high input resistance of these interneurons means that the current generated by a single quantum of glutamate released at a parallel fibre synapse can produce a significant shift in membrane voltage. Indeed, the action of a small number of coincident quanta appears sufficient to generate an action potential (Carter and Regehr, 2002). Thus, a relatively small change in the number or properties of glutamate receptor channels at parallel fibre-to-stellate/basket cell synapses, could influence interneuron activity and hence cerebellar output. In this respect it is of note that the prevalence of CP-AMPARs influences not only calcium influx, but also the amplitude, decay time and paired-pulse facilitation of synaptic currents, and hence the likelihood of action potential generation. Thus, a change in the contribution of synaptic CP-AMPARs has the potential to alter markedly neuronal circuit activity.

Although AMPA-, NMDA-, kainate- and metabotropic glutamate receptors (AMPARs, NMDARs, KARs and mGluRs) are all present in stellate cells (Fig. 1), the excitatory postsynaptic synaptic currents (EPSCs) generated by minimal simulation of parallel fibres, are mediated solely by AMPARs (Clark and Cull-Candy, 2002). A large proportion of synaptic AMPARs present in these cells are calcium-permeable (Liu and Cull-Candy, 2000), consisting predominantly of GluA3 homomers (Keinanen et al., 1990; Sato et al., 1993). However, GluA4 also appears to play some role in synaptic transmission as knocking-out GluA4 alters EPSC kinetics (Gardner et al., 2005). High frequency activity at these ‘calcium-permeable synapses’ produces a rapid alteration in AMPAR subtype from largely GluA2-lacking to GluA2-containing AMPARs that are less sensitive to intracellular polyamines and extracellular blockers such as Joro spider toxin and philanthotoxin-433 (PhTx-433) (Kelly et al., 2009; Liu and Cull-Candy, 2000). These observations provided an unequivocal demonstration of a functional switch in synaptic AMPAR subunit composition during a plasticity change, and identified a form of plasticity that appears to be relatively widespread in the CNS. This will be described in more detail below.

Of note, it is now clear that molecular layer interneurons also receive excitatory input from climbing fibres (Jorntell and Ekerot, 2003). In this case, direct synaptic contacts are not formed, but the cells sense overspill of glutamate from multiple climbing fibres (Mathews et al., 2012; Szapiro and Barbour, 2007). This unusual signalling involves activation of AMPARs, probably at extrasynaptic sites and parallel fibre contacts, and activation of extrasynaptic NMDARs.

Glutamate receptors are not restricted to the soma and dendrites of molecular layer interneurons, but are also found in axonal varicosities. This expression at GABA releasing terminals also appears to obey precise rules. Thus, while CI-AMPARs are expressed presynaptically at stellate cell-to-Purkinje cell contacts, stellate cell-to-stellate cell synapses express predominantly CP-AMPARs (Rossi et al., 2008; Rusakov et al., 2005). Activation of these presynaptic CP-AMPARs, by glutamate spillover from parallel- (Bureau and Mulle, 1998; Liu and Zukin, 2007) or climbing fibres (Rusakov et al., 2005; Satake et al., 2000), is thought to regulate the release of GABA.

### 2.2 CP-AMPARs in Bergmann glia and oligodendrocyte precursor cells

Bergmann glial cells – the radial glia of the cerebellar cortex – form processes that ensheath synaptic contacts made by climbing- and parallel fibres onto Purkinje cells (Fig. 1). As well as physically restricting the diffusion of glutamate, Bergmann glia actively remove it from the cleft *via* high affinity transporters (Bergles et al., 1997). Bergmann glia therefore play a key role in shaping of the glutamate waveform experienced by AMPARs at Purkinje cell synapses. In addition to the glutamate transporters, Bergmann glial cells also express CP-AMPARs (formed from GluA1 and GluA4) that are activated by transmitter spillover, and ectopic (quantal) release of transmitter from climbing and parallel fibre terminals (Bergles et al., 1997; Matsui et al., 2005).

It is clear that CP-AMPARs play a critical role in the regulation of the Bergmann glial cell's morphology (Iino et al., 2001) and transporter expression (Lopez-Bayghen et al., 2003). Indeed, rendering these AMPARs impermeable to calcium, by over expression of GluA2, causes Bergmann glia to retract their processes, which results in delayed clearance of glutamate from the synaptic cleft and abnormal synaptic transmission. Furthermore, when Bergmann glia express CI- instead of CP-AMPARs, the Purkinje cells remain innervated by multiple climbing fibres, rather than undergoing their normal developmental ‘pruning’ that results in innervation of mature Purkinje cells by just a single climbing fibre (Iino et al., 2001). Recently, complementary data have come from studies of inducible and astroglia-specific GluA1 and GluA4 double knockout mice, in which similar physiological and structural changes were accompanied by deficits in fine motor coordination (Saab et al., 2012). Glutamate activation of CP-AMPARs in Bergmann glia therefore appears to play an essential role in the development and maintenance of normal synaptic transmission at parallel- and climbing fibre inputs to Purkinje cells.

The nerve/glial antigen-2-positive oligodendrocyte precursor cell (NG2^+^-OPCs) is a major glial cell type that gives rise to myelinating oligodendrocytes throughout the brain. In the cerebellum, these cells are found in white matter and the molecular layer. Unlike Bergmann glia, they are not involved in the ensheathment of dendritic spines or removal of glutamate, rather they form discrete neuron-glia ‘synapses’ with climbing fibres. Transmission between climbing fibres and NG2^+^-OPCs shares many features with conventional synaptic transmission between neurons, including the presence of Ca^2+^-dependent transmitter release and activation of postsynaptic currents that are mediated by a mixture of CP- and CI-AMPARs (Lin et al., 2005).

The CP-AMPARs in NG2^+^-OPCs are of particular note, as they render these cells vulnerable to excitotoxic injury during early stages of development. Excessive calcium influx during ischemia triggers damage that can affect the ability of these cells to myelinate (Deng et al., 2006). CP-AMPARs in OPCs decrease during development and are absent in the mature oligodendrocytes. Interestingly the AMPARs present at synapses between climbing fibres and NG2^+^-OPCs also undergo a rapid activity-dependent plasticity. However, unlike the situation in stellate cells, EPSCs at neuron – NG2^+^ – synapses display an *increased* inward rectification following high frequency synaptic activity; this reflects a marked increase in the proportion of CP-AMPARs (Ge et al., 2006; Zonouzi et al., 2011). Although this difference between CP-AMPAR plasticity in stellate cells and NG2^+^ cells might seem profound, both forms of plasticity appear to share several common features (Zonouzi et al., 2011) that are considered below.

## 3 Mechanisms underlying CP-AMPAR plasticity in the cerebellum

Since the initial identification in cerebellar stellate cells of synaptic plasticity that involves a switch in the expression of AMPAR subtypes, from calcium-permeable to -impermeable (Liu and Cull-Candy, 2000), it has emerged that such dynamic changes in AMPAR GluA2-content occur widely throughout the CNS. Thus, the insertion and activation of CP-AMPARs, at normally ‘CI-AMPAR only’ synapses, seems to play a role in early stages of hippocampal LTP (Asrar et al., 2009; Lu et al., 2007; Plant et al., 2006; Yang et al., 2010). Other studies, using CP-AMPAR blockers, suggest that presence of CP-AMPARs is not required for the induction or maintenance of LTP at Schaffer collateral-to-CA1 pyramidal cell synapses (Adesnik and Nicoll, 2007; Gray et al., 2007). Therefore, this issue remains unresolved.

Importantly, changes in AMPAR subtype prevalence can occur during both physiological and pathological events and have been described in the lateral amygdala following fear conditioning (Clem and Huganir, 2010), in cortical neurons in response to sensory stimulation (Clem and Barth, 2006), in the ventral tegmental area of cocaine-treated animals (Bellone and Luscher, 2006), and in post-ischemic forebrain (Liu et al., 2004) and hippocampus (Noh et al., 2005). Our experiments, focussing mainly on cerebellar stellate- and NG2^+^ cells, have identified some of the mechanisms underlying CP-/CI-AMPAR plasticity and the auxiliary subunits involved in this process.

### 3.1 Switch in AMPAR subtype at parallel fibre-to-stellate cell synapses

**Fig. 2:**
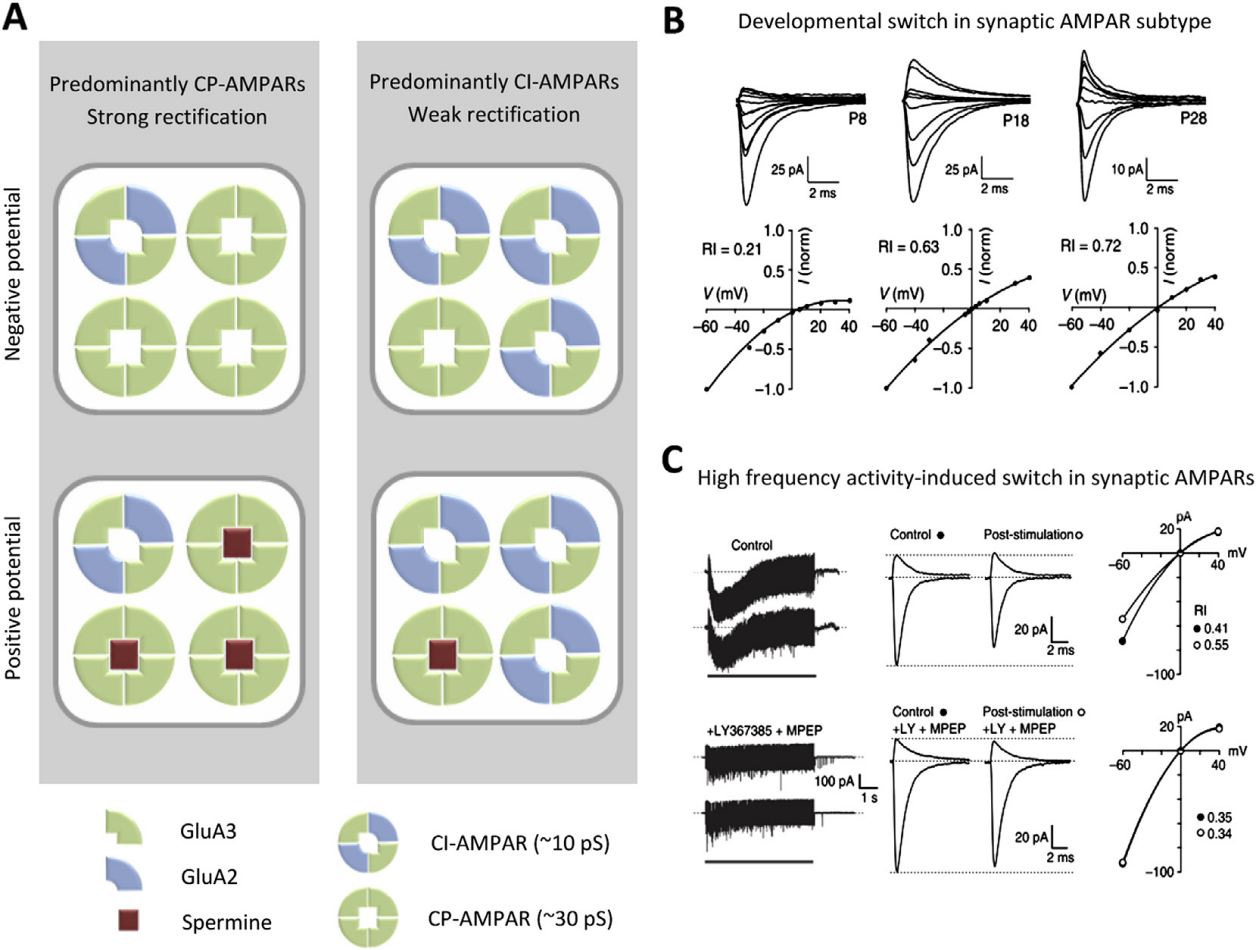
Developmental and activity-dependent changes in EPSC rectification reflect increased relative expression of CI-AMPARs at parallel fibre-to-stellate cell synapses. (A) Schematic diagram depicting how replacement of GluA2-lacking CP-AMPARs by GluA2-containing CI-AMPARs results in a decreased proportion of AMPARs blocked by intracellular spermine at positive potentials (strong *versus* weak rectification). Note that the single-channel conductance of CI-AMPARs is lower than that of CP-AMPARs. (B) Decreased AMPAR-mediated EPSC rectification during development. Recording made in slices from the cerebellum of 8, 18 and 28 day-old rats. Top panels, EPSCs evoked in stellate cells at membrane potentials ranging from −60 mV to +40 mV. Bottom panels, corresponding peak *I–V* relationships (RI; Rectification Index, calculated as conductance ratio: +40/−40 mV). Note in P8 stellate cells the strong current block at positive potentials, characteristic of CP-AMPAR expression. (C) High-frequency stimulation of parallel fibres is followed by a decreased EPSC rectification. Top panels from left to right: responses to a train of 100 stimuli at 50 Hz, averaged parallel fibre-evoked EPSCs at −60 and +40 mV in the same cell before and after high frequency stimulation, and corresponding *I–V* relationships. In the left-hand panels, note the fast AMPAR-mediated synaptic currents (and associated stimulation artefacts) together with mGluR-dependent slow currents. The switch from CP- to CI-AMPARs causes a decrease in the amplitude of EPSCs at negative potential (reflecting reduced single-channel conductance) and a reduction in rectification (reflecting increased spermine block at positive potentials). Bottom panels, same as top panels but in the presence of two mGluR antagonists. Note that the antagonists prevent both the slow current and the change in RI that follows the stimulation. (B and C, modified from Soto et al. (2007) and Kelly et al. (2009), respectively).

During cerebellar development, there is a gradual decrease in the contribution of CP-AMPARs to parallel fibre-to-stellate cell EPSCs (Soto et al., 2007) (Fig. 2A and B). However, even in the early postnatal cells where the overall proportion of CP-AMPARs can be very high, the relative level of expression of CI- and CP-AMPARs varies widely from cell to cell. The relative contribution of CI-AMPARs was found to be positively correlated with the level of spontaneous synaptic activity experienced by a cell (Liu and Cull-Candy, 2002, 2000). Consistent with the view that activity promotes a switch from CP- to CI-AMPARs at these synapses, high frequency stimulation of parallel fibres induces a rapid reduction in the inward rectification of EPSCs, indicative of an increased prevalence of CI-AMPARs (Fig. 2C) (Liu and Cull-Candy, 2000). Early studies demonstrated that calcium entry through the synaptic CP-AMPARs was required to trigger the removal of CP-AMPARs, and the incorporation of CI-AMPARs, at these synapses (Gardner et al., 2005; Liu and Cull-Candy, 2005, 2000) – suggesting the existence of a self-regulatory ‘feedback’ mechanism.

Paradoxically, under physiological conditions, depolarization of the postsynaptic membrane associated with high frequency activity would be expected to limit calcium influx, as a result of the enhanced block by intracellular polyamines and reduced ionic driving force. This might suggest other possible mechanisms also contribute to the required rise in intracellular calcium. Indeed, bath application of NMDA (Sun and June Liu, 2007), or of the group I mGluR agonist DHPG (Kelly et al., 2009), can both trigger a decrease in the relative proportion of CP-AMPARs at these synapses. This chemically induced plasticity is prevented by the inclusion of the calcium chelator BAPTA in the ‘intracellular’ (pipette) solution, indicating that an elevation of intracellular calcium level is required. Although surface NMDARs are not clustered at postsynaptic sites, and mGluRs are thought to be located perisynaptically (Nusser et al., 1994), both receptor types can be activated during high frequency synaptic activity (Clark and Cull-Candy, 2002; Karakossian and Otis, 2004) (Fig. 2C).

Indeed, there is compelling evidence to suggest group I mGluRs and NMDARs are likely to be activated during normal synaptic activity in these cells. Like most GABAergic interneurons, stellate cells lack synaptic spines, and parallel fibres form *en passant* synapses onto their dendritic shafts. While processes from Bergmann glia radiate throughout the molecular layer, they are sparse at stellate cell synapses and display only a low density of glutamate transporters, when compared with glial processes that ensheath parallel fibre-to-Purkinje cell connections (Chaudhry et al., 1995). Therefore, during high frequency bursts of parallel fibre activity, of the type that occurs in response to sensory input *in vivo* (Chadderton et al., 2004; Jorntell and Ekerot, 2006), transmitter is usually expected to reach extrasynaptic sites in the stellate cells (see Clark and Cull-Candy, 2002).

The plasticity evoked by high frequency stimulation of parallel fibre inputs to stellate cells has been examined in the presence of various AMPAR-, NMDAR- and mGluR blockers. The switch in AMPAR subtype is not inhibited by the NMDAR blocker d-AP5 (Liu and Cull-Candy, 2000), supporting the view that calcium influx through CP-AMPARs is sufficient to mediate the subunit switch. However, in circumstances where AMPARs are blocked during the induction protocol, activation of functional NMDARs is needed (Sun and June Liu, 2007). Indeed, while plasticity still occurs in the presence of an AMPAR antagonist, it is abolished when both NMDAR- and AMPAR-mediated currents are suppressed by the antagonists R-CPP and GYKI 52466, respectively.

Treatment with group I mGluR blockers (LY367385 and MPEP) also has a dramatic effect (Kelly et al., 2009). Not only do these drugs prevent the CP-AMPAR-dependent plasticity (triggered in the presence of d-AP5) (Fig. 2C), but their presence under resting conditions also increases the relative proportion of CP-AMPARs that contribute to normal transmission. Thus, under physiological conditions, tonic activation of group I mGluRs appears to set the ‘basal tone’ for CP-AMPARs at these synapses. It is of note that activation of mGluRs on their own is insufficient to trigger this form of synaptic plasticity, as no switch in AMPAR subtypes is observed when both AMPARs and NMDARs are blocked during the stimulation protocol (Sun and June Liu, 2007), or when calcium entry through AMPARs and NMDARs is blocked (Liu and Cull-Candy, 2000). Therefore, the combined action of ionotropic and metabotropic glutamate receptors at excitatory synapses in stellate cells is necessary and sufficient to trigger this form of plasticity (Kelly et al., 2009).

Taken together, these findings suggest that CP-AMPARs, NMDARs, and mGluRs act in concert to promote CI-AMPAR insertion at the parallel fibre-to-stellate cell synapses. However, it is worth noting that as activation of NMDARs requires transmitter spillover, the relative contribution of CP-AMPARs and NMDARs to the process is expected to vary depending on the pattern of presynaptic activity. Furthermore, as calcium diffusion within stellate cell dendrites is restricted by the presence of the calcium-binding protein parvalbumin and non-specific obstacles (Soler-Llavina and Sabatini, 2006), the precise location of the activated receptors is likely to be critical in their ability to trigger the plasticity.

Besides uncovering the role of glutamate in the control of synaptic AMPAR composition, these experiments on stellate cells implied an action potential-dependent modulation of CI-AMPARs expression (Liu and Cull-Candy, 2000). While most stellate cells in slices from juvenile rats fire action potentials at an average rate of around 10 Hz even in the absence of excitatory inputs (Häusser and Clark, 1997), a fraction of cells do not fire spontaneously. Strikingly, AMPAR-mediated currents evoked in patches excised from the soma of these non-spiking cells were more rectifying than average, suggesting that calcium entry through voltage-gated channels could also contribute to the surface expression of CI-AMPARs (Liu and Cull-Candy, 2000). Confirming this view, the relative contribution of CI-AMPARs to evoked currents was reduced in fast-spiking cells following treatment with TTX or calcium channels blockers (Liu and Cull-Candy, 2002, 2000). Conversely, blocking BK potassium channels, to prolong action potentials and consequently enhance calcium entry through L-type calcium channels, ultimately increased CI-AMPAR expression at these synapses (Liu et al., 2011). Such a change in action potential duration, under physiological conditions, also results in an increased contribution of CI-AMPARs to synaptic currents in these cells (Liu et al., 2010). The release of noradrenaline, in response to a fear-inducing stimulus, prolongs action potentials *via* activation of β-adrenergic receptors. This in turn enhances calcium entry through L-type calcium channels, triggering a sequence of events that lead to an intensification of GluA2 transcription. Accordingly, while the increase in CI-AMPARs caused by a presynaptic burst of activity, is restricted to one or few specific synapses, the increase in CI-AMPARs induced by such a prolongation of postsynaptic action potentials is likely to affect all synapses. The rise in GluA2 expression occurs over several hours, and results in a characteristic decrease in EPSC rectification and increase in decay time. However, this change in synaptic currents seems to be caused mainly by the addition of new CI-AMPARs to the synapse rather than a switch in AMPAR subtype.

### 3.2 Switch in CP-AMPAR subtypes: neuronal- and glial-specific rules

In OPCs the relative contribution of CP-AMPARs to AMPAR-mediated currents, can be rapidly altered by the activation of mGluRs or P2Y receptors (Zonouzi et al., 2011). In white matter, both neurons and astrocytes release glutamate and ATP. However, while activation of mGlu5 and P2Y receptors triggers a rise in intracellular calcium, the two transmitters have opposing effects on the relative expression of CP- and CI-AMPARs. The group I mGluR agonist DHPG promotes CP-AMPAR expression in OPCs, while expression is reduced by P2Y activation. Despite the striking difference between OPCs and stellate cells in their response to mGluR activation, the molecular mechanisms underlying DHPG induced plasticity in the two cell types show some clear similarities.

At stellate cell synapses, the removal of CP-AMPARs and the insertion of CI-AMPARs rely, respectively, on the disruption of GluA3 interaction with GRIP, and the binding of PICK1 to GluA2 (Gardner et al., 2005; Liu and Cull-Candy, 2005). While such interplay between specific AMPAR subunits and cytosolic PDZ domain-containing proteins was expected, our recent work shows that TARPs are also involved. Thus, in the absence of stargazin (TARP γ-2), the quantal synaptic events in stellate cells are mediated predominantly by CP-AMPARs displaying an increased block by both intracellular polyamines and extracellular PhTx-433. This suggests that while γ-2 may be required for GluA2-containing CI-AMPARs to reach the synapse, it is not essential for CP-AMPAR synaptic clustering (Bats et al., 2012). It is of note that another study argues against a selective impairment of GluA2 trafficking in *stg*/*stg* stellate cells (Jackson and Nicoll, 2011). While the authors of this work also observed an increased rectification of AMPAR-mediated currents in *stg*/*stg* stellate cells, they did not detect any associated increase in sensitivity to PhTx-433; the cause of this disparity is unclear. They thus concluded that the trafficking of both AMPAR subtypes is affected to the same extent by the lack of γ-2. However, it is clear that CP-AMPARs can still reach the synapse in the absence of γ-2 in stellate cells, or indeed in conditions where they lack any associated TARP (Bats et al., 2012).

A pool of extrasynaptic CI-AMPARs, together with CP-AMPARs, is normally present in the surface of stellate cells. Thus, in principle these would be readily available for recruitment to synapses. However, it remains to be determined whether this is the source of the new synaptic CI-AMPARs that are inserted following activity. In hippocampal neurons, it has been shown that freely diffusing GluA2-containing AMPARs can be trapped at a specific location in the plasma membrane, following a focal increase in the concentration of intracellular calcium (Borgdorff and Choquet, 2002). By avoiding exocytosis/endocytosis steps, the controlled clustering or dispersal of surface receptors would appear to represent a faster and more energy efficient way of adjusting AMPAR synaptic content. It has been proposed that CI-AMPARs can be delivered to stellate cell synapses through such a diffusion/trapping mechanism. However, it has been shown that DHPG-induced plasticity can be prevented by protein synthesis inhibitors (Kelly et al., 2009). This suggests that an increased expression of GluA2, or of proteins important in the differential trafficking of CP- and CI-AMPARs, is necessary. A rapid, activity-dependent alteration in the expression of CP-AMPARs has also been described at synapses in other regions of the brain. In dopaminergic neurons of the ventral tegmental area (VTA), cocaine induces the expression of CP-AMPARs that can be readily reversed by mGluR activation. In these VTA cells, the mGluR mediated synaptic insertion of CI-AMPARs relies on local synthesis of GluA2 (Mameli et al., 2007) and interaction with PICK1 (Bellone and Luscher, 2006), as seen in the stellate cells.

Unexpectedly, experiments on an OPC cell line (CG4 cells), and on optic nerve OPCs maintained in culture, have shown that many of the cellular mechanisms underlying the mGluR-induced plasticity of CP-AMPARs in OPCs resemble those seen in stellate cells (Zonouzi et al., 2011). Thus, the mGluR-induced switch in AMPAR subtypes not only requires protein synthesis, but also the interaction of the receptors with PICK1 and the binding of γ-2 to PDZ-domain containing proteins. Surprisingly, plasticity in stellate cells and OPCs both appear to involve similar cell processes and protein interactions, even though mGluR-activation results in opposite changes in synaptic transmission. Such variation in outcome could be explained by differences in the signalling pathways, in the subunit composition of AMPARs, in the assembly of the postsynaptic scaffold, or in the type of auxiliary proteins expressed in the two cell types.

In the hippocampus, activity-dependent changes in the contribution of CP- and CI-AMPARs to synaptic currents at Schaffer collateral contacts onto NG2^+^-OPCs provides another example of the physiological relevance of this form of plasticity. At these synapses, the switch in AMPAR subtype is triggered by theta stimulation. As in cerebellar NG2^+^-OPCs, a rise in intracellular calcium triggers an increase in synaptic CP-AMPARs (Ge et al., 2006). Whether such changes are developmentally restricted, are also seen at mature synapses, or can be induced by other stimuli, remains to be determined. Nevertheless, it seems possible that this difference in the activity-dependent switch in synaptic AMPAR subtypes – a decrease in CP-AMPARs in neurons *versus* an increase in OPCs – may be widespread in the CNS. In the case of OPCs in neonates, this may account for some of the pathological changes associated with ischaemia.

## 4 Role of TARPs in AMPAR trafficking, synaptic transmission, and plasticity in cerebellar cells

### 4.1 Synaptic transmission in the absence of conventional TARPs

The critical role played by TARPs in AMPAR expression was identified in cerebellar granule cells. Stargazin, γ-2, is enriched in the cerebellum where it associates with AMPARs, PSD-95, and related PDZ domain-containing proteins. In granule cells of *stg*/*stg* mice AMPAR surface expression is greatly impaired: the cells lack EPSCs and display little or no response to exogenous agonists (Hashimoto et al., 1999). As expected, transfecting *stg*/*stg* granule cells with wild-type γ-2 rescues both whole-cell and synaptic AMPAR-mediated currents. However, the clustering of AMPARs at synapses requires the γ-2 PDZ-binding motif. Thus, while expression of a mutant form of γ-2 lacking this specific C-terminal motif will rescue AMPARs surface expression in *stg*/*stg* granule cells, it fails to rescue the EPSCs (Chen et al., 2000).

Three further proteins, γ-3, γ-4 and γ-8, were later added to the TARP family, according to their ability to rescue AMPAR-mediated EPSCs when expressed in *stg*/*stg* granule cells (Tomita et al., 2003). Another two proteins, γ-5 and γ-7, closely related to the previously identified TARPs, have been classified as ‘atypical’ or ‘type II’ TARPs, as these fail to rescue EPSCs in *stg*/*stg* granule cells but interact with native AMPARs and alter their electrophysiological properties (Kato et al., 2008, 2007; Soto et al., 2009). In the cerebellum, while γ-5 expression is restricted to Bergmann glial cells and possibly NG2^+^-OPCs, γ-7 is present in most cells, including granule cells (see Fig. 1). Therefore, it is perhaps not surprising that transfection of granule cells with γ-7 (which is already present) failed to rescue their synaptic currents in *stg*/*stg*. However, as γ-7 is normally enriched at the synapse, where it interacts with AMPARs and PSD-95 (Kato et al., 2007; Yamazaki et al., 2010), the reason why endogenous γ-7 is unable to compensate for the absence of γ-2 remains unclear. Remarkably, not only does γ-7 fail to promote AMPAR synaptic expression in *stg*/*stg* granule cells, but it appears to have a suppressive action, as simple knock down of γ-7 rescues EPSCs, which are then mediated by TARPless AMPARs (Bats et al., 2012).

Additional observations, made in various cerebellar neurons, support the view that γ-7 is unable to maintain normal synaptic transmission in the absence of a conventional (type I) TARP (see Table 1). For example, in Purkinje cells from *stg*/*stg* mice, where γ-7 is likely to be the only TARP present, AMPAR-mediated synaptic currents are severely reduced (Menuz and Nicoll, 2008). However, currents mediated by extrasynaptic receptors in these cells appear normal. In Golgi cells, which ordinarily express γ-2, -3 and -7, the removal of both γ-2 and -3 induces a dramatic change in AMPAR-mediated EPSCs (Menuz et al., 2008). Although γ-7 is still thought to be present in the double knockout, the EPSC decay is faster, which might suggest the presence of TARPless AMPARs (as TARP-association normally prolongs EPSC decay time). Moreover, the *I–V* relationship of synaptic currents, which is usually linear in Golgi cells, becomes inwardly rectifying in γ-2/γ-3 double knockout mice, implying that the EPSCs are at least partially mediated by CP-AMPARs. It is of note that synaptic currents remain unchanged when γ-2 and γ-3 are knocked-out individually, suggesting that γ-2 and γ-3 may play somewhat similar roles.

Our recent work on *stg*/*stg* stellate cells indicates that the relative expression of CP-AMPARs is increased in the absence of γ-2 and that, under these conditions, stellate cell EPSCs are mediated mainly by CP-AMPARs (Bats et al., 2012); but see (Jackson and Nicoll, 2011). Furthermore, while the CP-AMPARs expressed in the extrasynaptic membrane of *stg*/*stg* stellate cells are associated with a TARP (γ-7), and are thus characterised by a high single-channel conductance, the mEPSCs in these cells are mediated by low conductance CP-AMPARs. This, and other evidence, strongly suggests that synaptic transmission in *stg*/*stg* stellate cells is mediated by TARPless CP-AMPARs. It therefore seems that the conventional TARPs, such as γ-2 or γ-3, may normally be required to promote not only the synaptic clustering of CI-AMPARs, but also that of γ-7-associated AMPARs.

Although transmission is altered, EPSCs are still present in *stg/stg* stellate- and Purkinje cells, and in γ-2/γ-3 knockout Golgi cells. This raises the question – why does the absence of a type I TARP (γ-2) affect transmission in granule cells to a greater extent than it does in other cerebellar neurons? The fact that γ-7 knock down rescues synaptic currents in *stg*/*stg* granule cells suggests its association with AMPARs may actively prevent them from accumulating at the synapse in the absence of other associated TARPs. As the relative expression of γ-7 and of AMPARs likely differs between neuron types, it is possible that, unlike in *stg*/*stg* stellate cells (or γ-2/γ-3 knockout Golgi cells), insufficient TARPless AMPARs are available for synaptic insertion and for maintenance of transmission in *stg*/*stg* granule cells.

Importantly, synaptic transmission in *stg/stg* stellate cells, and in Golgi cells that lack γ-2/γ-3, appears to be mediated mainly or entirely by CP-AMPARs. Stellate cells from wild-type mice normally express a proportion of CP-AMPARs, and Golgi cells are clearly capable of expressing these when γ-2 and -3 are absent. On the other hand, it is unclear whether granule cells can normally express any CP-AMPARs. So this may account for the absence of transmission in *stg/stg* granule cells. Interestingly, low levels of synaptic GluA4 have been detected in *stg*/*stg* granule cells (Chen et al., 2000; Yamazaki et al., 2010). While these receptors are insufficient to generate detectable EPSCs, small AMPAR-mediated whole-cell currents have been described (Kato et al., 2007). Determining whether these arise from CI- or CP-AMPARs would certainly be of interest. It is also of note that the overexpression of γ-7 in *stg*/*stg* granule cells enhances the whole-cell currents (Kato et al., 2007; Milstein et al., 2007), although the underlying mechanism remains unclear. For example, it is not known whether the increased current arises from a change in γ-7/AMPAR stoichiometry (which could potentially increase the receptors' channel conductance), or from an increase in the number of AMPARs present at the cell surface.

The selective and dramatic effect on granule cells of γ-2 loss may also reflect the fact that the assembly and composition of the postsynaptic scaffold varies between different types of neurons. For example, it has been shown that the sequence of events leading to the synaptic clustering of AMPARs and NMDARs differs between pyramidal and aspiny neurons of the hippocampus (Mi et al., 2004). In pyramidal neurons, the synaptic clustering of AMPARs requires the interaction of conventional TARPs with postsynaptic PSD95, whereas clustering of NMDARs involves their direct binding to PSD-95. By contrast, in hippocampal aspiny interneurons, the synaptic expression of AMPARs is independent of the interaction of γ-2 (or related TARPs) with PSD-95. Indeed, in these cells the AMPARs recruit PSD-95 to the synapse *via* their associated TARPs, and thereby enable the clustering of synaptic NMDARs. Thus, differences in the contribution of cytosolic, extracellular and/or transmembrane AMPAR-interacting proteins to the assembly of the postsynaptic density could explain why certain neurons are more susceptible to the absence of conventional TARPs.

### 4.2 Role of TARPs in the differential trafficking of CP- and CI-AMPAR subunits

Dramatic changes are observed in the synaptic expression of AMPARs in cerebellar neurons lacking γ-2 (Table 1). AMPAR expression in the cerebellum of γ-7 knockout mice is also markedly altered, even though γ-7 knockout mice do not appear phenotypically very different from wild-type animals (Yamazaki et al., 2010). Importantly, the decrease in AMPARs that is caused by specific deletion of either γ-2 or γ-7 is not uniform. Rather, loss of either of these TARPs appears to affect a different set of AMPAR subunits. Namely, loss of γ-2 causes a significant decrease in GluA2, GluA3 and GluA4 in cerebellar extracts (with GluA2 and GluA3 being the most reduced). On the other hand, loss of γ-7 decreases mainly GluA1 and GluA4 subunits. Hence, by controlling the level of specific AMPAR subunits, and thus the composition of AMPAR complexes, γ-2 and γ-7 could, in principle, regulate the relative expression of CI-AMPARs and CP-AMPARs in the cerebellum.

Immunofluorescent labelling of AMPAR subunits has demonstrated that the decrease in GluA1 and GluA4 in the cerebellum of γ-7 knockout mice is, in large part, due to loss of AMPARs in Bergmann glia (Yamazaki et al., 2010). Interestingly, both γ-5 and γ-7 are normally present in these cells, and there is functional evidence that γ-5 is normally associated with CP-AMPARs (Soto et al., 2009). However, it is clear that γ-5 cannot compensate for the absence of γ-7 in knockout animals. Together these data suggest that both of these type II TARPs may be required for normal function of CP-AMPARs in Bergmann glial cells.

In addition to its effects on AMPARs in Bergmann glia, knockout of γ-7 also induces a marked decrease in synaptic GluA4 in granule cells. As these cells normally express only CI-AMPARs, such increase in the relative proportion of GluA2 would not be expected to cause any change in calcium permeability. On the other hand, knockout of γ-7 is expected to affect the ratio of CP-/CI-AMPARs at parallel fibre synapses in stellate cells. Indeed, γ-7 knockout selectively disrupts GluA3 synaptic expression in stellate cells. As GluA3 forms homomeric CP-AMPARs in these cells, one would predict a decrease in the prevalence of CP-AMPARs at the synapse. Therefore, while functional data indicate that conventional TARPs (such as γ-2) are required for the normal expression of CI-AMPARs, immunofluorescent studies, immunogold labelling, and functional data all suggest that γ-5 and γ-7 may affect the expression of CP-AMPARs. Such an arrangement favours the view that the relative expression of CP- and CI-AMPARs in the cerebellum is regulated by the combined action of type I and type II TARPs.

### 4.3 Role of TARPs in CP-AMPAR-dependent plasticity

While γ-2 and other conventional TARPs promote the expression and synaptic accumulation of AMPARs in various cerebellar neurons, recent evidence indicates that they also play a critical role in AMPAR plasticity in cerebellar cells. This has been examined in stellate cells (Bats et al., 2012; Jackson and Nicoll, 2011) and OPCs (Zonouzi et al., 2011). Although high frequency stimulation of parallel fibres normally triggers a decrease in EPSC rectification in wild-type stellate cells, a recent study suggests that in *stg*/*stg* stellate cells EPSC rectification remains unaltered following activity (Jackson and Nicoll, 2011). However, the interpretation of this observation is complicated by that fact that, in this particular study, the basal level of CP-AMPARs appeared to be identical at wild-type and *stg*/*stg* stellate cell synapses, as there was no significant difference in EPSC sensitivity to PhTx-433, a selective blocker of CP-AMPAR channels. This finding is at odds with subsequent experiments that indicate a greatly increased level of CP-AMPARs at *stg*/*stg* stellate cell synapses, as signified by an increase in PhTx-433-mediated block of mEPSCs (Bats et al., 2012). In addition, although an activity-dependent decrease in EPSC rectification was observed in wild-type stellate cells, it did not correlate with a reduced sensitivity to PhTx-433 (Jackson and Nicoll, 2011).

Our work previously demonstrated that TARPs partially relieve the block of AMPARs by endogenous intracellular polyamines (Soto et al., 2007), thereby decreasing the inward rectification of synaptic currents mediated by CP-AMPARs. The extent of the relief varies between TARPs, being greatest for ‘typical’ TARPs and least for ‘atypical’ family members (γ-5 and γ-7) (Soto et al., 2009). It has been suggested (Jackson and Nicoll, 2011), that rather than reflecting a switch from CP- to CI-AMPARs, the plasticity at parallel fibre-to-stellate cell synapses could be ascribed merely to the replacement of AMPARs that were strongly blocked by polyamines (TARPless, or TARPed with γ-7), with ones that were weakly blocked (γ-2-containing) and therefore less rectifying. However, this seems unlikely as stellate cells treated with the mGluR agonist DHPG (which replicates the effects of high frequency activity), exhibit EPSCs that are less rectifying and also display a clear-cut reduction in PhTx-433 block (Kelly et al., 2009). The latter observation can only easily be accounted for by a reduction in CP-AMPARs. Thus, while it is apparent that plasticity at parallel fibre-to-stellate cell synapses involves a switch from CP- to CI-AMPARs, the role played by γ-2 remains to be defined. However, there is compelling evidence that CP-AMPARs can still reach the synapse in the absence of γ-2, or indeed in conditions where the CP-AMPARs are TARPless (Bats et al., 2012). While this refutes the dogma that only TARPed AMPARs can mediate EPSCs, it seems unlikely that this is a situation that normally pertains *in vivo*.

Although group I mGluR activation in stellate cells triggers a decrease in EPSC rectification, it has the opposite effect in OPCs (causing an increase in the prevalence of CP-AMPARs). In these cells, transfection with a mutant form of γ-2 lacking the carboxy-terminal PDZ binding motif causes a shift in the *I–V* relationship of AMPAR-mediated currents, from inwardly rectifying to linear, and importantly, prevents the DHPG-induced plasticity (Zonouzi et al., 2011). Therefore, it seems that the interaction of γ-2 (or related TARPs) with endogenous PDZ domain-containing proteins is required to specifically deliver and/or retain CP-AMPARs at the surface of OPCs, and hence to control the relative contribution of CI- and CP-AMPARs to excitatory currents in these cells.

## 5 Conclusion

The dynamic regulation of CP-AMPARs plays a key role in the physiology of various neurons and glial cells in the central nervous system. Based on recent findings in the cerebellum, TARPs are involved not only in canonical forms of plasticity such as hippocampal LTP and LTD (Tomita et al., 2005b), and LTD in Purkinje cells (Nomura et al., 2012), but are also implicated in CP-AMPAR plasticity. While γ-5 appears to play a specific role in CP-AMPAR delivery (Soto et al., 2009; but see Kato et al., 2008), it will be interesting to determine whether the other type II TARP γ-7 shows similar selectivity. However, other important questions also remain to be addressed, including: (1) how do TARPs contribute to the differential expression of CP- and CI-AMPARs beyond the cerebellum, (2) what are the molecular mechanisms accounting for the apparent opposite action of γ-2 in the regulation of CP-AMPAR expression in neurons and glia, (3) how do type I and type II TARPs cooperate to regulate the relative expression of CP- and CI-AMPARs, and (4) are other auxiliary subunits also involved in this process? Indeed, while finalizing this review, a study appeared showing that CNIH-2/-3 (Cornichon-2/-3, another group of AMPAR auxiliary subunits) selectively promotes the trafficking of GluA1-containing receptors to the plasma membrane of hippocampal neurons (Herring et al., 2013). Interestingly, this subunit-specific action appears to rely on the prevention of the interaction of CNIH with non-GluA1 subunits by TARP γ-8 (which is enriched in hippocampus). This shows that a CNIH/TARP interplay is involved in the subunit-specific trafficking of AMPARs, and by extension suggests that interaction between different auxiliary subunits could also be involved the control of the expression of specific AMPAR subtypes, such as CP- and CI-AMPARs.

## Figures and Tables

**Table 1:** Evidence of TARP involvement in the differential expression of CP- and CI-AMPARs in cerebellar cells. References: [a] (Menuz et al., 2008); [b] (Chen et al., 2000); [c] (Yamazaki et al., 2010); [d] (Bats et al., 2012); [e] (Jackson and Nicoll, 2011); [f] (Zonouzi et al., 2011). Abbreviations: KD, knock down; MF, mossy fiber; PF, parallel fiber; CF, climbing fiber; Ct, C-terminal; dKO, double knockout; KO, knockout.

Cell type (main AMPAR subunits)	AMPARs		TARPs		Effect on EPSCs or AMPAR immunolabelling	CI-/CP-ratio
	CI-	CP-	Normal content	Manipulation		
Golgi cells (GluA2, 3)	✓		γ-2, -3 and -7	γ-2/3 dKO	EPSCs become rectifying [a]	↓
Granule cells (GluA2, 4)	✓		γ-2 and -7	*stg*/*stg* or γ-2 KO γ-7 KO γ-7 KD in *stg*/*stg*	Loss of EPSCs [b] Loss of GluA2 labelling, trace of synaptic GluA4 remains [b, c] Selective reduction of GluA4 labelling at MF synapses [c] EPSCs are rescued [d], but see [c]^a^	↓ = =
Purkinje cells (GluA1, 2, 3)	✓		γ-2 and -7	*stg*/*stg* or γ-2 KO	Decrease in EPSC amplitude at both CF and PF synapses [d] Marked reduction of GluA2 labelling at CF synapses [b]^b^	?
Stellate cells (GluA2, 3)	✓	✓	γ-2 and -7	*stg*/*stg* γ-7 KO	Increased contribution of CP-AMPARs to EPSCs [d] Lack of CP-AMPAR plasticity [e]^c^ Selective reduction of GluA3 labelling at PF synapses [c]	↓ ↑
Bergmann glia (GluA1, 4)		✓	γ-4, -5 and -7	γ-7 KO	Loss of both GluA1 and 4 labelling [c]	?
Oligodendrocyte precursors (GluA1-4)	✓	✓	γ-2 (-3, -4 and -5)	Dominant negative γ-2ΔCt	Increased contribution of CI-AMPARs to EPSCs and lack of CP-AMPAR plasticity [f]^d^	↑

a EPSCs are rescued by the acute shRNA-mediated knock down of γ-7 in *stg*/*stg* granule cells (Bats et al., 2012). However, in γ-2/γ-7 double knock out granule cells, the clustering of GluA4 at synapse is further reduced compared to γ-2 knock out (Yamazaki et al., 2010). While the reason for this discrepancy is unclear, it is of note that, unlike in transgenic γ-2/γ-7 double knock out animals, the knock down of γ-7 was acute and affected only transfected cells.

b Of the four AMPAR subunits normally present in Purkinje cells, GluA2 expression at climbing fibre synapses is the most affected by γ-2 knock out. However, the *I*–*V* relationship of AMPAR-mediated whole-cell currents remains linear (Yamazaki et al., 2010). This suggests that even if AMPARs at climbing fibre-to-Purkinje cell synapses are permeable to calcium, they represent only a small fraction of surface AMPARs.

c In this study (Jackson and Nicoll, 2011), the authors argue that the increase in EPSC rectification in *stg*/*stg* stellate cells is not associated with a decrease in the contribution of CI-AMPARs, and hence does not reflect a change in the CI-/CP-AMPAR ratio at the synapse.

d Experiments were carried out in OPCs cultured from optic nerve.
